# Skeletal Muscle Phosphodiester Content Relates to Body Mass and Glycemic Control

**DOI:** 10.1371/journal.pone.0021846

**Published:** 2011-07-14

**Authors:** Julia Szendroedi, Albrecht Ingo Schmid, Marek Chmelik, Martin Krssak, Peter Nowotny, Thomas Prikoszovich, Alexandra Kautzky-Willer, Michael Wolzt, Werner Waldhäusl, Michael Roden

**Affiliations:** 1 Institute for Clinical Diabetology, German Diabetes Center, Leibniz Center for Diabetes Research, Heinrich-Heine University Düsseldorf, Düsseldorf, Germany; 2 Department of Metabolic Diseases, Heinrich-Heine University Düsseldorf, Düsseldorf, Germany; 3 Karl-Landsteiner Institute for Endocrinology and Metabolism, Medical University of Vienna, Vienna, Austria; 4 MR Center of Excellence, Medical University of Vienna, Vienna, Austria; 5 Department of Radiology, Medical University of Vienna, Vienna, Austria; 6 Department of Internal Medicine 3, Medical University of Vienna, Vienna, Austria; Mayo Clinic, United States of America

## Abstract

**Background:**

Aging and insulin resistance have been related to reduced mitochondrial function and oxidative stress. Muscular phosphodiesters (PDE) are comprised of metabolites of phospholipid breakdown and may reflect membrane damage. We aimed to test the hypothesis that myocellular PDE are increased in patients with type 2 diabetes (T2D) and correlate inversely with mitochondrial ATP turnover.

**Methods:**

A Cross-sectional study in the Clinical Research Facility of an University hospital was performed. 10 nonobese middle-aged patients with T2D, 10 healthy humans matched for sex, age and physical activity index (CONm) and 18 young healthy humans (CONy) were included. Myocellular PDE and unidirectional flux through ATP synthase (fATP) were measured with ^31^P magnetic resonance spectroscopy (MRS). Intramyocellular (IMCL) and hepatocellular lipid deposition (HCL) were quantified with ^1^H MRS. Insulin sensitivity (Rd) was assessed from hyperinsulinemic-euglycemic clamp tests in 10 T2D, 10 CONm and 11 CONy.

**Results:**

During fasting, T2D and CONm had 1.5 fold greater PDE than CONy (2.8±0.2, 2.5±0.2, 1.7±0.1 mmol/l, P = 0.004). Stimulation by insulin did not affect PDE in any group. PDE correlated negatively with Rd (r = −0.552, p<0.005) and fATP (r = −0.396, p<0.05) and positively with age (r = 0.656, p<0.001) and body mass (r = 0.597, p<0.001). PDE also related positively to HbA1c (r = 0.674, p<0.001) and fasting plasma glucose (r = 0.629, p<0.001) within T2D and across all participants.

**Conclusions:**

Muscular PDE concentrations associate with age, lower resting mitochondrial activity and insulin resistance, which is determined mainly by body mass and glycemia.

## Introduction

Type 2 diabetes mellitus (T2D) represents one of the world's greatest economic and health care challenges. T2D results from an imbalance between insulin responsiveness and insulin secretion. Skeletal muscle is mainly responsible for whole-body insulin resistance and determines substrate oxidation particularly during exercise. Reduced mitochondrial oxidative capacity, activity and/or content have been related to aging, intramyocelluar lipid content (IMCL) and muscular insulin resistance [1], [2], [3], [4].

It has been hypothesized that aging-associated insulin resistance results from cumulative free radical damage leading to lower mitochondrial function and increased IMCL [5]. Of note, preventing oxidative damage by overexpression of antioxidant defense mechanisms indeed protected rodent models from lipid-induced and age-associated insulin resistance [5], [6]. However, a causal relationship between aging, mitochondria and the development of T2D has been questioned [1], [7], [8]. Of note, there is no evidence for disruption of the cellular integrity and contractile function in skeletal muscle of T2D, while patients with myopathies can exhibit mitochondrial abnormalities and insulin resistance [9], [10], [11].

Membrane phospholipids are hydrolyzed to free fatty acids (FFA) and phosphodiesters (PDE) which are regarded as cell membrane degradation products [12]. Myocellular PDE are elevated in patients with myopathies [13], [14] and in elderly [15], [16] and increase in response to treatment with statins [17]. PDE may reflect fiber atrophy, accumulated sarcolemmal damage [18] or structural and functional changes during loss of muscle mass possibly resulting from cumulative oxidative damage [19]. Thus, PDE may serve as marker of biomembrane integrity [18], [20]. The relationship between PDE content and mitochondrial function and insulin sensitivity in healthy middle-aged humans and in patients with T2D has not yet been reported.

We hypothesized that T2D have elevated myocellular PDE which relates to age, mitochondrial activity and insulin sensitivity. Thus, we measured PDE, unidirectional flux through ATP synthase (fATP) during fasting and insulin stimulation as a marker of resting mitochondrial activity [21], [22], insulin stimulated glucose-6-phosphate (ΔG6P) as a marker of insulin-stimulated glucose transport/phosphorylation, whole-body glucose disposal (Rd) and endogenous glucose production (EGP) in T2D, non-diabetic age-matched (CONm) and younger humans (CONy).

## Methods

### Ethics Statement

All studies were carried out in accordance with the most-recent version of the Declaration of Helsinki and approved by the local ethics committee of the Medical University of Vienna. Informed written consent has been obtained from all participants.

### Subjects

Ten T2D, 10 CONm matched for sex, age and physical activity and 18 CONy were included. PDE content in all participants from one study [22] and 7 controls from another study [23] are reported. They underwent complete medical history, clinical examination and lab tests. All participants had comparable physical activity according to Baeckés questionnaire, refrained from any physical exercise for three days and fasted for 12 h before the study. T2D had neither islet cell antibodies nor signs of neurological disorders including diabetes-related neuropathy. Only sulfonylurea and/or metformin were allowed as glucose-lowering medication but withdrawn three days before the study. Four T2D and none of the healthy participants received statins, which were not withdrawn before the study. Control subjects had no family history of T2D.

### Experimental Protocol

After baseline blood sampling, D-[6,6-^2^H_2_]glucose (98% enriched; Cambridge Isotope Laboratories, http://www.isotope.com/cil/index.cfm) was given as primed-continuous infusion [0 min to 5 min: 3.6 mg.(kg body weight)^−1^.(fasting plasma glucose in mg/dl)/(90 mg/dl); −115 min to +240 min 0.036 mg.min^−1^.(kg body weight)^−1^] to assess Rd and EGP from +220 to +240 min. From 0 to +240 min, hyperinsulinemic-euglycemic clamp tests were performed in 10 T2DM, 10 CONm and 11 CONy, while 7 CONy were only examined at baseline. Insulin (Actrapid; Novo, Bagsvaerd, Denmark) was administered as primed-continuous infusion [40 mU.(m body surface area)^−2^.min^−1^] and plasma glucose was controlled by a variable 20% dextrose infusion, 2% enriched with D-[6,6-^2^H_2_]glucose according to the hot-glucose-infusion protocol.

IMCL, PDE and other phosphorus metabolites were measured at baseline and during insulin stimulation.

### Magnetic Resonance Spectroscopy (MRS)

**Figure 1:**
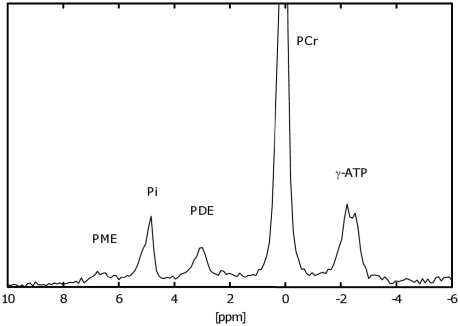
Representative ^31^phosphorous magnetic resonance spectrum of skeletal muscle. Phosphorus compounds were measured from the ratio of the integrated respective peak intensities and ß-ATP resonance intensity in spectra without inversion and saturation (pulse length 150 µs/90°, 2k data, 8 averages, repetition time of 15 s) assuming a constant ATP concentration of 5.5 mmol/l muscle.

Measurements were performed on subjects lying supine inside a 3-Tesla MR spectrometer (Medspec S300-DBX; Bruker, Ettlingen, Germany). The right lower leg positioned on a 10-cm circular double resonant ^1^H/^31^P surface coil (125.6/50.8 MHz) so that the isocenter of the magnetic field was placed ∼2 cm into the medial head of the gastrocnemius muscle [22]. Phosphorus compounds were measured from the ratio of the integrated peak intensities and ß-ATP resonance intensity in spectra without inversion and saturation (pulse length 150 µs/90°, 2k data, 8 averages, repetition time of 15 s) assuming constant ATP concentrations of 5.5 mmol/l muscle [22]. In human skeletal muscle, the peak between inorganic phosphate (Pi) and phosphocreatine (PCr) represents the signal arising from PDE which is mostly attributed to glycerophospho-ethanolamine/-choline, membrane-bound phospholipid metabolites (**Figure 1**) [24]. Measurement of PDE has been validated against chemical analysis employing preparations of muscular ethanol-soluble PDE and exogenous glycerol-3-phosphorylcholine, which confirmed that MRS-measured PDE represent the peak resonating at 0.13 ppm [24].

Myocellular fATP [µmol.(g muscle ^−1^).min^−1^] was measured with ^31^P MRS employing the saturation transfer experiment to the Pi/ATP exchange [22], [25]. Glucose transport/-phosphorylation was assessed from the increase in G6P during the clamp (ΔG6P). IMCL in soleus muscle and liver fat content (HCL) were measured by ^1^H MRS [22], [26].

### Analytical measurements

Plasma glucose was assessed by the glucose oxidase method (Beckman, Fullerton, CA), FFA microfluorimetrically (Wako Chem. USA Inc., Richmond, VA) and plasma insulin by double-antibody radioimmunoassay (Pharmacia, Uppsala, Sweden). All other measurements were assessed in the routine lab.

### Calculations and statistics

At baseline, rates of glucose appearance (R_a_) were calculated by dividing the tracer D-[6,6- ^2^H_2_]glucose infusion rate times tracer enrichment by the percent of tracer enrichment in plasma and subtracting the tracer infusion rate [27]. During the clamp, R_a_ was calculated using Steele's non-steady state equations [28]. EGP is given as the difference between R_a_ and mean glucose infusion rates.

Group data are presented as means and SD (Text) or SEM (Figures) and compared with ANOVA and Tukey post-hoc testing as appropriate. Within-group differences were assessed with two-tailed t-tests. Linear correlations are Pearson product-moment correlations. Partial correlation analysis was performed to study the linear relationship between PDE and other variables after excluding the effect of age, BMI or HbA1c. Differences were considered significant at the 5-% level.

## Results

All groups were matched for sex (50% female). BMI tended to be (T2D: 27±3; CONm: 26±3; CONy: 24±2 kg/m^2^) and waist circumference was higher in T2D and CONm (97±9; 92±17; 80±8 cm, p<0.05 T2D vs. CONy). T2D and CONm were also matched for age (59±6; 57±7; 29±5 years, p<0.001 both vs. CONy). T2D had greater fasting plasma glucose (8.9±1.7; 5.2±0.4; 4.8±0.7 mmol/l, p<0.001 vs. both CON groups) and HbA1c (6.9±0.7; 5.5±0.3; 5.2±0.2 mmol/l, p<0.001 vs. both CON), but comparable plasma FFA (0.57±0.11; 0.44±0.22; 0.48±0.26 mmol/l) and low-density lipoprotein (LDL: 3.4±0.8; 3.8±0.9; 2.6±0.5 mmol/l).

During clamp steady-state (220–240 min), mean plasma glucose, insulin and FFA were 5.5±0.5 mmol/l, 514±96 pmol/l and 0.02±0.01 mmol/l without differences between groups. T2D had ∼33% and ∼54% lower Rd than CONm and CONy; CONm had ∼31% lower Rd than CONy (5.7±0.5, 8.5±0.8, 12.4±1.0 mg.kg^−1^.min^−1^, p<0.05, p<0.001 T2D vs. CONm and CONy, p<0.005 CONm vs CONy). Insulin-suppressed EGP reflecting hepatic insulin resistance was greater in T2D and CONm (0.23±0.05; 0.22±0.09; −0.14±0.09 mg.kg^−1^.min^−1^, p<0.05 both vs. CONy).

Resting mitochondrial activity (fATP) was lower in T2D than in CONy but comparable to CONm [22]. Stimulation by insulin increased fATP in CONm and CONy but not in T2D and unmasked impaired mitochondrial adaptation compared to CONm [22].

**Figure 2:**
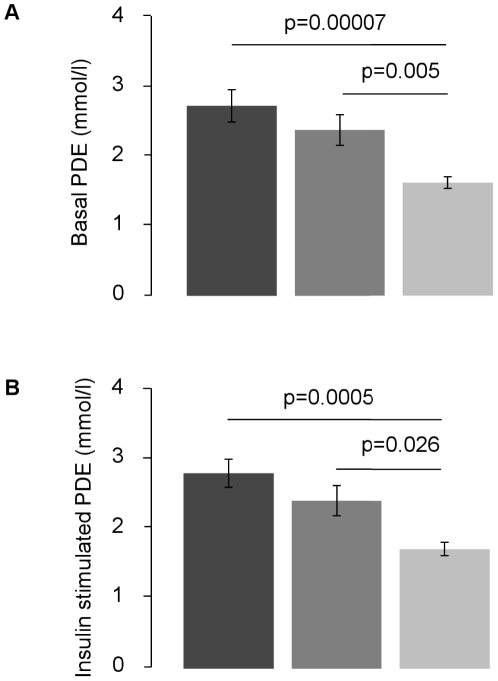
Phosphodiester (PDE; means ± SEM) content in 10 patients with type 2 diabetes (T2D, black columns), 10 age-matched (CONm, grey columns) and 18 lean young controls (CONy, empty columns) during fasting (A) and during insulin stimulation (B).

In the fasted state, myocellular PDE contents were ∼65% and ∼47% higher in T2D and CONm compared to CONy, but not different between T2D and CONm (**Figure 2A**). IMCL and ΔG6P were comparable in all groups as reported [22] and did not relate to PDE. Insulin stimulation did not affect PDE (basal vs. insulin-stimulated: 2.8±0.8 vs. 2.9±0.7; 2.5±0.7 vs. 2.5±0.7; CONy: 1.8±0.3 vs. 1.8±0.3 mmol/l), which were ∼55% and ∼38% higher in T2D and CONm compared to CONy (p<0.001, p<0.05, **Figure 2B**). Changes in PDE did not relate to ΔG6P.

**Figure 3:**
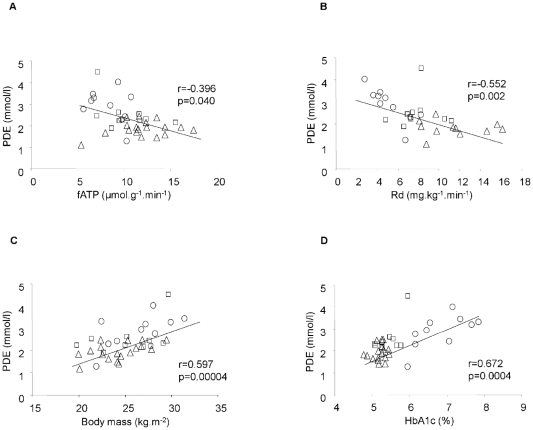
Association between fasting myocellular phosphodiester (PDE) contents and flux through ATP synthase (fATP) (A), whole-body glucose disposal (Rd) (B), body mass index (BMI) (C) and glycemic control (HbA1c) (D) in 10 patients with type 2 diabetes (T2D, circles), 10 age-matched (CONm, squares) and 11–18 lean young controls (CONy, triangles) during fasting.

PDE correlated negatively with fasting fATP (**Figure 3A**) and insulin-mediated Rd (**Figure 3B**), but not with suppressed EGP or insulin-stimulated fATP. PDE related positively to BMI (**Figure 3C**), waist circumference (r = 0.668, p<0.001), age (r = 0.656, p<0.001), HCL (r = 0.446, p<0.01), HbA1c (**Figure 3D**) and to fasting plasma glucose (r = 0.629, p<0.001) across all participants and within T2D (r = 0.652, r = 0.653, p<0.05). There was no correlation between muscle PDE and HbA1c in the pooled group of non-diabetic subjects. PDE neither related to physical activity nor to plasma FFA, LDL or IMCL. Correlations of PDE with fATP and Rd were abolished after selective adjustment for either age, BMI or HbA1c.

## Discussion

Myocellular PDE levels are higher in middle-aged patients with T2D and in age-matched controls than in young non-diabetic humans. The relationships of PDE with insulin resistance and mitochondrial function seem to depend mainly on aging, body mass, but also glycemia.

The greater PDE in the middle-aged humans with or without T2D is in accordance with its positive relationship to age as reported previously [16], but confounding factors such as insulin resistance or mitochondrial function were not identified before [18]. Aging-related alterations of skeletal muscle could simply result from reduced voluntary physical activity, but PDE - in line with previous data [29] - did not relate to physical activity index. All groups of the present study were sedentary and matched for physical activity, but exercise testing was not performed. PDE has been shown to be increased in professional road cyclists [30], but to be significantly lower in sprinters than in sedentary and long-distance runners [31]. Accordingly, a large PDE signal has been reported as a sign of a larger relative content of type 1 fibers, i. e. fast-to-slow twitch muscle transformation [32]. However, this observation does not fit with increased PDE in aging or diabetic subjects, who rather have increased [33], [34], [35] or normal fast glycolytic fiber fractions [36], [37]. Also, statins have been shown to increase muscle PDE contents [17], but only four of our patients with T2D were on statins and their PDE content was not different from the other patients. Alternatively, aging-associated cumulative oxidative damage may affect mitochondrial DNA and function and lead to muscular degenerative processes [38]. Our T2D showed lower fATP than both control groups during insulin stimulation, while resting fATP was similarly lower in T2D and CONm than in CONy [22].

In vivo and ex vivo measures reported lower oxidative capacity in exercising and resting muscle of T2D patients which result from intrinsic impairment and lower mitochondrial content [3], [4], [39], [40], [41]. fATP, reflecting demand-driven resting mitochondrial activity, is lower in insulin-resistant elderly [21], [22] and non-diabetic offspring of T2D patients [42], but can be normal in T2D compared to carefully matched non-diabetic humans [22], [43]. Alternatively, abnormalities in insulin signaling may be the primary events leading to impaired mitochondrial function, or both phenomena may be mutually interrelated [44]. Of note, lower mitochondrial content could be compensated for by greater mitochondrial activity suggesting that insulin sensitivity and mitochondrial function are not uniformly coupled [8], [45], [46], [47]. Nevertheless, partial correlation analysis revealed that PDE contents relate to aging independently of BMI and physical activity, but not independently of fATP and insulin sensitivity. Thus, the impact of aging on PDE is likely mediated by insulin sensitivity and fATP.

Partial correlation analysis further identified PDE contents as an independent indicator of glucometabolic control (HbA1c and fasting plasma glucose) in T2D. Elevated PDE as observed in muscle damage [17], pain syndromes [29] and muscle dystrophies, has been previously attributed to oxidative stress [18], [20], [48]. One might therefore speculate that glucotoxicity-induced chronic oxidative stress could contribute to the greater PDE as well as to the lower fATP and insulin sensitivity in our T2D. This study found a weak negative relationship between muscular PDE and fATP, which disappeared upon adjusting for age, BMI and HbA1c. While this does not exclude any role of mitochondrial damage for raising PDE levels, it makes such mechanism rather unlikely. However, fATP is only one feature of mitochondrial function and in the present study mitochondrial content and oxidative capacity were not measured. Furthermore, fATP as assessed from the ATP saturation transfer experiment with ^31^P MRS, reflects in vivo ATP synthase flux resulting from basal energy demand and supplying processes and is therefore a measure of resting mitochondrial activity. [49].

Muscular PDE related negatively to whole-body glucose disposal but not specifically to insulin-mediated glucose transport/phosphorylation and did not change during insulin stimulation even in insulin sensitive humans. While PDE do not seem to directly reflect insulin-dependent metabolic processes, its tight relationship with BMI points to obesity-associated alterations such as fatty acid composition of membrane phospholipids which may influence insulin binding and action [50]. While lipid-induced insulin resistance generally arises from increased plasma FFA and intracellular lipids [51], this study found no association between PDE, FFA and IMCL. Nevertheless, PDE are generated by increased activities of phospholipases [16] which also yield ceramides and diacylglycerols (DAG), possible mediators of insulin resistance [52], [53].

Some limitations need to be taken into account. First, no biopsies were taken so that identification of individual components of the PDE peak is not possible. However, the analysis of individual compounds contributing to the PDE signal is difficult, because ischemia during tissue handling will impact on analysis of extracted tissue [24]. Second, no indicator of oxidative stress was determined to analyse the possible role of PDE contents as a biomarker of oxidative stress. However, available markers of oxidative stress rely on various assumptions and do not offer direct measures [6], [54]. Finally, our T2D cohort comprised of normal weight (n = 4), overweight (n = 4) and obese (n = 2) Caucasians yielding a mean BMI of 27 kg/m^2^, which was not significantly different but tended to be higher than in controls. While T2D cohorts mostly have mean BMI exceeding 30 kg/m^2^, other T2D cohorts have lower mean BMI values, e. g. 28.5 kg/m^2^ in the ADVANCE study [55]. Thus, our T2D group might be suitable and representative to analyse the impact of T2D per se on PDE contents.

In conclusion, muscular PDE are higher in middle-aged patients with or without T2D than in young non-diabetic humans. Body mass and glycemia mainly determine the correlations of PDE with resting mitochondrial activity, insulin resistance and age.
